# Environmental Factors-Induced Oxidative Stress: Hormonal and Molecular Pathway Disruptions in Hypogonadism and Erectile Dysfunction

**DOI:** 10.3390/antiox10060837

**Published:** 2021-05-24

**Authors:** Shubhadeep Roychoudhury, Saptaparna Chakraborty, Arun Paul Choudhury, Anandan Das, Niraj Kumar Jha, Petr Slama, Monika Nath, Peter Massanyi, Janne Ruokolainen, Kavindra Kumar Kesari

**Affiliations:** 1 Department of Life Science and Bioinformatics, Assam University, Silchar 788011, India; chakrabortysaptaparna@gmail.com (S.C.); anandandas852@gmail.com (A.D.); monikanath111@gmail.com (M.N.); 2 Department of Obstetrics and Gynecology, Silchar Medical College and Hospital, Silchar 788014, India; drarunpc@gmail.com; 3 Department of Biotechnology, School of Engineering and Technology (SET), Sharda University, Greater Noida 201310, India; niraj.jha@sharda.ac.in; 4 Department of Animal Morphology, Physiology and Genetics, Faculty of AgriSciences, Mendel University in Brno, 61300 Brno, Czech Republic; petr.slama@mendelu.cz; 5 Department of Animal Physiology, Faculty of Biotechnology and Food Sciences, Slovak University of Agriculture in Nitra, 94976 Nitra, Slovakia; Peter.Massanyi@uniag.sk; 6 Department of Applied Physics, School of Science, Aalto University, 00076 Espoo, Finland; janne.ruokolainen@aalto.fi (J.R.); kavindra.kesari@aalto.fi (K.K.K.)

**Keywords:** hypogonadism, erectile dysfunction, testosterone, infertility, pesticide, radiation, air pollution, heavy metals, endocrine-disrupting chemicals

## Abstract

Hypogonadism is an endocrine disorder characterized by inadequate serum testosterone production by the Leydig cells of the testis. It is triggered by alterations in the hypothalamic–pituitary–gonadal axis. Erectile dysfunction (ED) is another common disorder in men that involves an alteration in erectile response–organic, relational, or psychological. The incidence of hypogonadism and ED is common in men aged over 40 years. Hypogonadism (including late-onset hypogonadism) and ED may be linked to several environmental factors-induced oxidative stresses. The factors mainly include exposure to pesticides, radiation, air pollution, heavy metals and other endocrine-disrupting chemicals. These environmental risk factors may induce oxidative stress and lead to hormonal dysfunctions. To better understand the subject, the study used many keywords, including “hypogonadism”, “late-onset hypogonadism”, “testosterone”, “erectile dysfunction”, “reactive oxygen species”, “oxidative stress”, and “environmental pollution” in major online databases, such as SCOPUS and PUBMED to extract relevant scientific information. Based on these parameters, this review summarizes a comprehensive insight into the important environmental issues that may have a direct or indirect association with hypogonadism and ED in men. The study concludes that environmental factors-induced oxidative stress may cause infertility in men. The hypothesis and outcomes were reviewed critically, and the mechanistic approaches are applied through oxidant-sensitive pathways. This study also provides reccomendations on future therapeutic interventions and protective measures against such adverse environmental factors-induced hypogonadism and ED.

## 1. Introduction

Hypogonadism is an endocrine disorder characterized by inadequate serum testosterone production by the Leydig cells of the testis [1]. It occurs due to the disruption of the normal functioning of the hypothalamic–pituitary–gonadal (HPG) axis, which subsequently alters the functioning of Leydig cells in the testis [2]. It not only hampers the process of spermatogenesis but also disturbs normal reproductive physiology. Associated symptoms include decreased energy, reduced libido, weight gain and reduction in muscle mass in the male [1]. Some epidemiological studies showed that community-dwelling men above the age of 30 years are characterized by an annual decrease of 0.5–15% in the concentration of circulating testosterone and a 2–3% decrease in the concentration of free testosterone [3]. Around 40% of men above 45 years and 50% of men above 80 years of age have been found to be hypogonadal [4]. Sometimes considered as the male equivalent of female menopause, the age-related clinical and biochemical syndrome in men is also termed as andropause [5], late-onset hypogonadism (LOH) [6], symptomatic late-onset hypogonadism (SLOH) [7], male climacteric, viropause, androgen deficiency in aging males (ADAM) [8], partial androgen deficiency in aging male (PADAM), and testosterone deficiency syndrome (TDS) [9]. In men, a high prevalence of hypogonadism has been reported with life and work stresses [10]. Another common male sexual disorder is ED that involves an alteration in any of the components of erectile response–organic, relational, and psychological [10]. It is also common in men aged over 40 years [11], and more than half of these men have been reported to be afflicted by ED [10]. Psychological, physiological, hormonal, pathological, environmental, and nutritional stressors are believed to play a major role in the pathogenesis of ED [10]. An alteration in any one or combination of these factors may lead to the disease [12]. In terms of endocrine factors leading to ED, reduced serum testosterone levels have been implicated. Still, the exact mechanism is yet to be elucidated fully [13]. The majority of men with hypogonadism, ED and/or other chronic illness are clustered with high numbers in the age group of 40–60 years [10,14]. Hence, identification of roles of various mediators of normal gonadal and erectile functions is imminent in understanding the pathogenesis of both hypogonadism and ED, wherein reactive oxygen species (ROS) acts as a common mediator.

ROS are oxygen-containing reactive molecules present in the body. They are maintained at an optimum concentration by various antioxidant enzymes. However, high ROS levels can disrupt the oxidative balance, thereby impacting the HPG axis resulting in reduced secretion of reproductive hormones [15]. Oxidative stress occurs either due to enhanced production of ROS or reduced availability of antioxidants. ROS play an important role as second messengers in many intracellular signaling cascades for maintaining cellular homeostasis with its immediate environment. During oxidative stress, biomolecules undergo indiscriminate damage, leading to loss of function and even cell death. Being highly reactive oxygen-containing free radicals, they extract lone pairs of electrons from the nearby biomolecule, leading to its inactivity, which results in a cascade of molecular damages [16]. ROS may cause lipid peroxidation in the Leydig cells and germ cells, damage to lipoproteins, protein aggregation and DNA fragmentation, as well as inhibition of steroidogenic enzymes [17]. Testicular oxidative stress causes a reduction in testosterone production, either because of the injury to the Leydig cells or to other endocrine structures like the anterior pituitary [18,19]. Testosterone is known to regulate ED by stimulating nitric oxide (NO), releasing pathways in cavernosal tissues. NO upregulates cyclic guanosine monophosphate (cGMP) levels required for the increased blood flow during erection [20]. Interaction between NO and ROS is one of the important mechanisms implicated in the pathophysiological process of ED [21]. NO interacts with superoxide to form peroxynitrite, which inactivates superoxide dismutase (SOD) and leads to an increased amount of superoxide. Peroxynitrite-mediated induction of superoxide leads to endothelial dysfunction and reduction in NO levels leading to ED. The ROS-mediated reaction of superoxide and NO results in acute impairment of cavernosal relaxation and induces long-term penile vasculopathy. ROS have been implicated in both neurogenic ED and vasculogenic ED [22]. Environmental factors, such as pesticides, radiation, air pollutants, agents originated from plastics, and other endocrine disruptors, are important contributing determinants of hypogonadism and ED. These stressors follow several mechanisms, including stimulation of ROS production, which, in turn, creates a state of oxidative stress in the reproductive tissues leading to the above-mentioned andrological problems. Pesticides induce oxidative stress that results in lipid peroxidation and DNA damage, which also reduces testosterone levels [23]. Radiations from different sources enhance the generation of seminal ROS, which leads to oxidative stress, too [24]. Exposure to various air pollutants, plasticizers and endocrine-disrupting chemicals also enhances producing ROS in the body that affects the male reproductive system [25,26,27]. The objective of the current evidence-based study was to reveal the association of environmental factors, such as pesticides, radiation, air pollutants, and other endocrine-disrupting chemicals, including agents originating in plastics in the pathogenesis of hypogonadism and ED. We have also attempted to provide a comprehensive insight into the probable mechanisms, particularly the oxidant-sensitive pathways through which these stressors exert their pathological impacts.

## 2. Methods

Electronic database Scopus was selected for the search of studies on the literature. The following keyword strings and Boolean operators were used for the Scopus search: TITLE-ABS (“hypogonadism” OR “late-onset hypogonadism” OR “testosterone” OR “erectile dysfunction”) AND TITLE-ABS (“reactive oxygen species” OR “ROS” OR “oxidative stress”) AND TITLE-ABS (“pesticide” OR “chlorpyrifos” OR “diazinon” OR “cypermethrin” OR “radiation” OR “ray” OR “mobile” OR “cell^*^ phone” OR “radar” OR “air pollution” OR “cadmium” OR “lead” OR “particulate matter” OR “PM2.5” OR “bisphenol A” OR “phthalate^*^” OR “perfluoroalkyl^*^” OR “PFAS” OR “polychlorinated biphenyl” OR “PCB”). A total number of 385 articles were identified by applying the keyword search strategy. The articles identified were subsequently screened manually by title, keywords and abstract for eligibility. Only the original articles and reviews in the English language were included after using the automatic filters present in the database. Those papers, which were not in English or whose translations were not found, were excluded along with other types of publications, such as conference papers, book chapters, short surveys, letters, notes, editorials, and books, leaving 31 articles as excluded. The studies on human and rodent models were included, and the studies on lower organisms were rejected. Through manual screening of the title, keywords and abstract, 140 non-relevant articles were excluded. Full-text articles were then reviewed for eligibility using the inclusion and exclusion criteria, resulting in 214 articles that were eligible for inclusion. However, 89 articles from other publicly available sources, such as PUBMED, MEDLINE and World Health Organization, were also included. They contained important information pertaining to oxidative stress induced by environmental factors and their impact on hypogonadism and ED. Thus, a total of 303 articles was finally included in the present study from the above-mentioned search.

## 3. Testosterone Metabolism, Spermatogenesis, and Reactive Oxygen Species (ROS)

The manifestation of male reproductive wellbeing is dependent on the coordinated functioning of testosterone, and its regulation is an essential prerequisite for the process of spermatogenesis [28]. The initiation point of testosterone biosynthesis is the hypothalamus, which secretes gonadotropin-releasing hormone (GnRH). In turn, GnRH stimulates producing luteinizing hormone (LH) and follicle-stimulating hormone (FSH) from the pituitary. Subsequently, LH is transported in the bloodstream to the testes, where it stimulates the Leydig cells to produce testosterone [29]. Testosterone and LH initiate the functional responses required to support spermatogenesis by binding to the androgen receptor present on the Sertoli cells, Leydig cells, peritubular myoid cells, arteriole smooth muscle cells and vascular endothelial cells [30]. However, ROS interfere with the communication between the Leydig cells and the HPG axis, lowering testosterone levels [15]. Excessive generation of ROS can further stimulate the hypothalamic-pituitary-adrenal (HPA) axis, releasing cortisol. Through the crosstalk between the HPG and HPA axes, cortisol negatively affects the LH secretion from the anterior pituitary, which is followed by decreased testosterone production by the Leydig cells. Testosterone synthesis is also dependent on the concentration of sialic acid present in Sertoli and Leydig cells, and decreased sialic acid level negatively impacts the testosterone concentration [31,32]. Henceforth, it may be hypothesized that a decline in sialic acid concentration impacts the Leydig cells, and ROS may play a role in downregulating sialic acid levels. Severe oxidative stress also reduces FSH release, which reduces the generation of androgen-binding protein (ABP) from the Sertoli cells, further declining the levels of circulating testosterone [15]. Regulation of testicular functioning and spermatogenesis is dependent on the number and control of Sertoli cells [33]. Sertoli cells often fall victim to various environmental stressors and endocrine-disrupting-chemicals-induced oxidative stress, which has been associated with disturbance in hormonal secretions, blockage of gap junctional communications, and damage to the blood–testis barrier integrity [34,35,36,37,38].

In addition to the HPG axis-mediated endocrine functioning, intragonadal paracrine and autocrine factors also converge in a complex stage-specific multifactorial control of spermatogenesis [39]. These include various locally secreted peptides and hormones, such as growth hormone (GH), insulin-like growth factor-1 (IGF-1), cytokines, activin, inhibin, follistatin and estrogen [40]. GH acts both directly and indirectly via the hepatic IGF-1, at the testicular level, to promote sperm production. It is also expressed in the testicular tissues, which regulates local processes that are strategically regulated by pituitary GH. GH promotes the early development of spermatogonia and ensures the complete maturation of germ cells [41]. The balanced endocrine interplay of the HPG axis is under the feedback control of another pituitary hormone called prolactin or luteotropic hormone (LTH). LTH controls the secretion of LH and FSH via regulating GnRH in the hypothalamus. Maintenance of physiological levels of serum LTH is critical in mediating the process of spermatogenesis [42]. Inhibin is a negative regulator of FSH secretion, and excessive release of this hormone inhibits the normal spermatogenic process. During the condition of oxidative stress, ROS tends to stimulate producing inhibin, along with testicular estradiol (E2), which results in reduced testosterone synthesis [43]. ROS exposure also upregulates the secretion of LTH, which decreases GnRH release. Additionally, enhanced oxidative stress stimulates generating proinflammatory cytokines, such as tumor necrosis factor-alpha (TNF-α), interleukin-1 beta (IL-1β) and interleukin-6 (IL-6), which negatively affects the HPG axis, thereby downregulating testosterone biosynthesis [15]. Therefore, a fine interplay of the HPG axis as well as other paracrine and autocrine factors is essential not only for producing male germ cells but also for the preservation of male reproductive functions.

**Figure 1:**
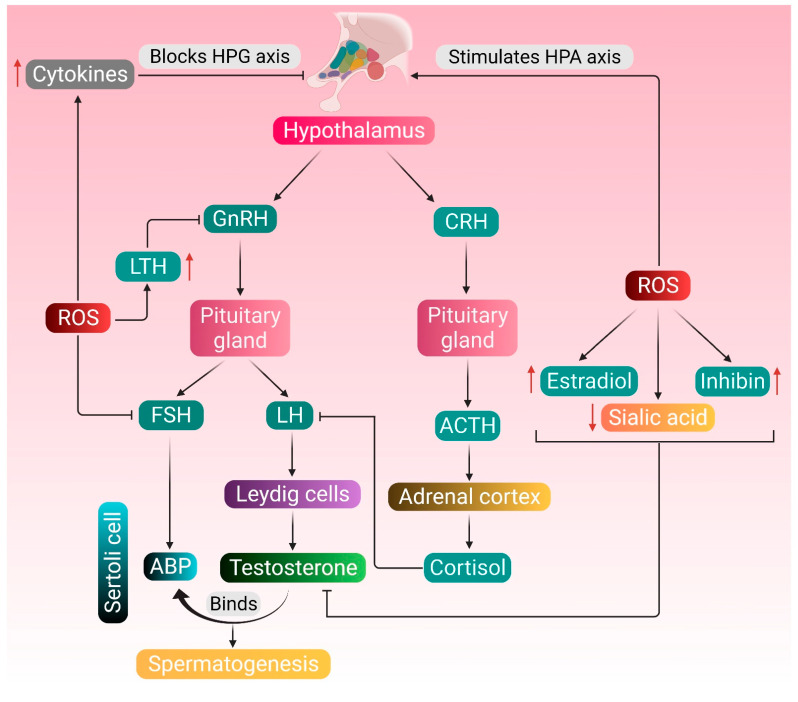
Endocrine regulation of testosterone metabolism and spermatogenesis, and the possible attack points of ROS-induced pathologies. Normal physiology of testosterone metabolism and spermatogenesis is regulated by the hormones secreted by the hypothalamus, pituitary gland and Leydig cells. The pituitary gland produces LH and FSH in response to GnRH secreted by the hypothalamus. LH stimulates the Leydig cells to produce testosterone, and FSH upregulates the production of ABP in the Sertoli cells. Testosterone binds to ABP and brings about the functional response required for spermatogenesis. ROS stimulates the HPA axis, thereby mediating the adrenal cortex to synthesize cortisol, which inhibits the action of LH. ROS also upregulate the production of inhibin and estradiol and downregulates the concentration of testicular sialic acid—all these negatively affect the action of testosterone. Increased production of LTH resulting from excessive ROS generation may also create hindrance in the HPG axis. ROS may directly affect the HPG axis by inhibiting the production of FSH or stimulate the generation of cytokines, such as TNF-α, IL-1β and IL-6, which in turn block the HPG axis. Red arrows represent the increase and decrease of respective substances, which negatively affects the HPG axis. (GnRH: gonadotropin-releasing hormone; LH: luteinizing hormone; FSH: follicle-stimulating hormone; ABP: androgen-binding protein; CRH: corticotropin-releasing hormone; ACTH: adrenocorticotropic hormone; LTH: luteotropic hormone; TNF-α: tumor necrosis factor-alpha; IL-1β: interleukin- 1 beta; IL-6: interleukin 6; HPG: hypothalamic–pituitary–gonadal; HPA: hypothalamic–pituitary–adrenal; ROS: reactive oxygen species).

Endocrine regulation of testosterone metabolism and spermatogenesis and the possible attack points of ROS-induced pathologies are shown in Figure 1.

## 4. Etiology and Pathogenesis

With the increase in age, the level of sex-hormone-binding globulin decreases, resulting in lower levels of testosterone available for some tissues in elderly men [44,45]. In addition, serum levels of testosterone decline through pituitary (central) and testicular (peripheral) mechanisms [46]. Furthermore, it is believed that the blood concentration of male hormones may not represent the amount of hormonal activity, which is influenced by the function of the hormone itself [5]. As mentioned earlier, testosterone deficiency is associated with oxidative stress, which decreases the amount of testosterone in the body via lipid peroxidation, DNA damage or protein modifications [23].

Adults with pituitary insufficiency have been found to be deficient in producing normal concentrations of pituitary hormones, including gonadotropins, and the condition usually results in testosterone deficiency at some point [47]. This condition is also likely to disrupt the normal maintenance of sperm concentration in such patients. Low levels of circulating testosterone are likely to lower the sperm count. However, the combined effect of intratesticular testosterone and FSH is often sufficient to induce normal spermatogenesis. Moreover, adult hypogonadic men have been documented with a significant lowering of motile spermatozoa than normal individuals [48]. With passing age, sperm concentration, motility and morphology decrease each year [49,50]. Pronounced semen volume drops have also been noted among men over 45 years [51]. Other parameters, such as prostate-specific antigen (PSA), fructose, glucosidases and zinc levels, have been found to be lower in men above the age of 50 [52]. Moreover, a study conducted on middle-aged and aging Chinese men with hypogonadism reported a high incidence of ED. Particularly, the effect of LOH symptoms on ED further decreased their testosterone levels [53]. Low testosterone levels have been associated intricately with symptoms, such as poor morning erection, low libido, ED, inability to perform vigorous activity, depression and fatigue in a group of men aged 40–79 years [54].

### 4.1. Hypogonadism

Hypogonadism is of two types—hypergonadotropic or primary hypogonadism and hypogonadotropic or secondary hypogonadism. The main cause of primary hypogonadism is congenital, including Klinefelter syndrome, anorchia, enzyme defects in the androgen synthesis, and cryptorchidism [6]. Individuals with Klinefelter syndrome display a spectrum of gonadal failures, with most patients going undiagnosed until adulthood [55]. Anorchia is a genetic condition defined as the absence of testis in individuals with 46 XY phenotype and may manifest prenatal testicular vascular accident associated with torsion during testicular descent [56]. Cryptorchidism or the failure of testicular descent in the scrotum may lead to malfunctioning of the testes and reduced production of testosterone [57]. Another important cause of primary hypogonadism is acquired, including orchitis, testicular torsion, castration, cytotoxic therapy, and normal aging. Hypogonadotropic or secondary hypogonadism is caused mainly due to hypothalamic dysfunctions or anomalies in the pituitary gland functioning [6,58]. Problems associated with hypogonadism could be due to one or more factors that go inappropriate in their functioning in the HPG axis [59].

In men, hypogonadism may lead to the absence of secondary sexual characteristics, infertility, muscular dystrophy, and other abnormalities [58]. In primary hypogonadism, the testes are mainly affected by a surge in the serum LH and FSH levels. In such conditions, reproductive organs receive signals from the brain to produce sex hormones. Still, due to abnormality in the gonad itself, sufficient testosterone production cannot be achieved, resulting in hypogonadism [59]. Secondary hypogonadism is regarded as central hypogonadism because it can be identified with the cerebrum motioning to the gonad. LH and FSH levels are partially normal or are low in most cases [59,60]. With the increment in age, there is a characteristic decrease in androgen generation due to the impairment of LH and FSH generation. It subsequently results in decreased synthesis of testosterone, leading to hypogonadism. Although the condition is common, the exact cause has remained a matter of investigation [59]. Hemochromatosis, an iron overload disease, is another causal factor of secondary hypogonadism [61]. Surplus of iron in the body may have deleterious effects on endocrine functioning, particularly on the pituitary axis, which is considered a common origin of secondary hypogonadism [62]. This may also lead to testicular oxidative stress, subsequently causing sperm DNA damage, lipid peroxidation and protein alterations [63].

Hypogonadism is considered as the most important clinical feature of LOH– a term coined in 2002 and specified by the International Society of Andrology (ISA), International Society for the Study of the Aging Male (ISSAM), European Urology (EAU), European Academy of Andrology (EAA), and American Society of Andrology (ASA) [64]. The typical symptoms include (i) changes in physical characteristics (abdominal obesity, decreased muscle mass and strength, decreased bone mineral density, decreased body hair, and skin alterations), (ii) changes in mood (depression, decreased concentration and cognitive function, and increased fatigue), and (iii) decreased sexual function (diminished libido and erectile quality) [5].

### 4.2. Erectile Dysfunction (ED)

In a normal adult, penile erection is mediated by the neurotransmitter (NO) via cGMP. Phosphodiesterase type 5 inhibitors PDE-5I inhibit the hydrolysis of cGMP into GMP, causing increased NO to relax smooth muscle and increase blood flow, thereby facilitating erectile response [65]. Neurogenic, vasculogenic, iatrogenic and endocrine pathways are considered responsible for the occurrence of ED [13]. Neurogenic ED encompasses neurologic disorders, which may act centrally, peripherally, or both. It is characterized by the suppression of NO release from the nerves in the corpora cavernosa, which ultimately results in ED [66]. Vasculogenic ED is most commonly caused by inadequate levels of cGMP, resulting in decreased calcium ion concentration. This, in turn, results in lower blood flow in the cavernous tissues, consequently leading to reduced penile erection [67]. Iatrogenic ED arises due to various medical and surgical therapies, which tend to disrupt the normal conduction of complex events necessary for achieving a penile erection [68]. Testosterone is an important hormone in regulating erectile function, which enhances neurogenic and endothelial NO synthesis. Thus, a disruption in testosterone regulation may give rise to endocrine-related ED [69]. Therefore, a complex interaction of physiological, vascular, neural, and endocrine factors is critical to erectile functions. A negative impact in any of these pathways may result in ED [70]. The 2013 updateby ISSAM advises screening of LOH in men having symptoms of ED [71].

ED has been associated with a loss of meaning and value of a man’s life leading to psychological frustration, feelings of helplessness and shame, family disagreement, social conflict, and lower self-confidence, ultimately exerting an adverse effect on couple intimacy and sex life harmony [72,73]. ED has a positive correlation with age, having an incidence of about 6–15% at the age 40–49 years, 19–22% at 50–59 years, 30–44% at 60–69 years, and 37–70% among men of ≥70 years age [74,75,76]. Aged men having other complications, especially those suffering from diabetes, are more likely to develop ED [77]. As mentioned earlier, reduced blood testosterone level is an indicator of andropause. It is also a leading causal factor of ED. Thus, hypogonadism and/or LOH symptoms are often accompanied by the reduced capability of the penile erection [78]. Although ED does not affect life expectancy, in most populations, it appears to have a negative effect on overall well-being [13].

## 5. Environmental Factors

Certain environmental, lifestyle and metabolic factors have been associated with the symptoms of hypogonadism (including LOH) and ED in men through many pathways [79]. Pesticides, radiation (including cell phone usage), air and water pollution, other endocrine-disrupting chemicals, smoking, consuming alcohol and illicit drugs, obesity and stress, are believed to contribute to disease development [79,80,81]. Environmental toxins, radiation, certain drugs and medications can affect the healthy functioning of testes [82]. A positive association between air pollution and ED has also been reported [83].

### 5.1. Pesticides

Pesticide pollution is known to affect the male reproductive system. Humans are generally exposed to pesticides through food and intake of vegetables and fruits with elevated residual pesticide concentration that can cause depletion of sperm count in men. There are several such pesticides, which can exert negative effects on male reproductive functions. Imidacloprid or 1-(6-chloro-3-pyridylmethyl)-N-nitroimidazolidin-2-ylideneamine, an aminoacetal pesticide used in modern agricultural practices, has been found to interfere with the levels of LH, FSH and testosterone, thereby causing TDS [84]. Dichloro-diphenyl-trichloroethane (DDT), another commonly used pesticide, possesses anti-androgenic properties and causes hormonal disturbances in the body characterized by hypogonadism [85]. Testosterone concentration can decrease in men after acute exposure to pesticides, such as carbamates, thio- and dithiocarbamates, pyrethroids, chlorophenoxy acids, chloromethylphosphoric acids, and organophosphatases [86]. Pesticides like diazinon can decrease spermatozoa motility and morphology [87]. Bendiocarb administration can disrupt spermatogenesis by impairing the ultrastructure of rabbit spermatozoa. However, the damage gets slowly repaired after the excretion of the pesticide [88].

A study was conducted on farmers exposed to pesticides regularly. The exposure period of the farmers to different pesticides ranged from 2 to 11 years. Monitoring for 6 months revealed decreased levels of serum testosterone and LH [89]. Low testosterone level can also be attributed to low LH level through feedback mechanism [90]. Pesticides can change the pathway of steroid synthesis by interacting with the plasma membrane and nuclear receptors by imitating sex hormones, such as androgens [91]. Diazinon, chlorpyrifos and cypermethrin has been found to exert negative consequences on testosterone production, which tends to disrupt overall men’s health. These pesticides bring about hormonal dysfunction by stimulating producing ROS and induction of oxidative stress. The harmful organophosphate pesticides also tend to disrupt molecular mechanisms required for normal erectile functioning through induction of oxidative stress. They are involved in decreasing the glutathione buffer and increasing the levels of superoxide, which reacts with the penile NO and hampers the achievement of normal erection. The concentration of NO decreases due to its reaction with superoxide, and subsequent formation of peroxynitrite may lead to ED [21]. Moreover, superoxide tends to upregulate the mobilization of Ca^2+^ in blood, resulting in the constriction of the penile smooth muscles, thereby reducing a man’s ability to achieve penile erection [86]. Currently, the following are considered biomarkers of ROS-induced vascular endothelial dysfunction—insulin resistance, homocysteinemia, lipoprotein A, eNOS inhibitors, vasodilators, such as prostaglandins, adhesion molecules like vascular adhesion molecule 1 (VCAM-1), intracellular adhesion molecule 1 (ICAM-1), selectins and thrombic homeostatic factors [92]. Made up of endothelial cells, the endothelium is responsible for producing NO through the eNOS pathway. Negative effects on any of these markers may contribute to the pathophysiological process leading to ED [86]. From the hormonal perspective, ROS-induced testosterone reduction can disrupt proper maintenance of NOS activity, due to which NO is reduced, ultimately giving rise to ED [93].

In a study, male mice were injected with 30 mg/kg body weight of organophosphorus pesticide diazinon for five consecutive days a week for one month. The results showed a reduction in the diameter of seminiferous tubules, the number of spermatocytes and serum testosterone levels [23]. Diazinon has also been found to alter the activity and biosynthetic pathway of steroidogenic hormones, including testosterone, which leads to the impairment of normal reproductive physiology [23,94]. Diazinon is most likely to interrupt testosterone production by disrupting the transmission of endocrine neurons in the hypothalamus, followed by an imbalance of the HPG axis [95]. Enhanced diazinon exposure has also been correlated with increased serotonin concentration in the anterior hypothalamus, which is one of the main reasons for the decline in LH and FSH concentrations, possibly resulting in the decrease of testosterone levels [95]. In addition, this organophosphate pesticide has a stimulatory effect on LTH release along with a hyperprolactinemic state, which is often associated with low LH levels [96]. Furthermore, chronic exposure to diazinon impairs Leydig cell functions, which is displayed by decreased testosterone production, apparently due to reduced expression of several important steroidogenic factors, including steroidogenic acute regulatory protein (StAR), cytochrome P450 17A1 (CYP17A1), cytochrome P450 11A1 (CYP11A1) and 3β-hydroxysteroid dehydrogenase (3β-HSD) [97]. Diazinon can also decrease serum testosterone levels by enhancing lipid peroxidation, DNA damage, and changes in antioxidant enzyme action [23]. Diazinon exposure has been found to decrease glutathione and catalase levels, which, together with lipid peroxidation, may lead to the induction of oxidative stress in the Leydig cells [98]. The subsequent decline in testosterone levels may disrupt NOS activity in the penile tissues, further suppressing NO production, ultimately giving rise to ED [86]. Hence, it may be hypothesized that diazinon exposure may result in diminished erectile functions.

In another study, organophosphorus pesticide chlorpyrifos, when administered to rats at 7.5, 12.5 and 17.5 mg/kg bodyweight for 30 days, has been found to decrease the serum testosterone levels together with a decline in sperm count and motility. Chlorpyrifos markedly decreased the testicular sialic acid and glycogen levels, too, while increasing the level of cholesterol [31]. Chlorpyrifos-induced decrease in sialic acid content in testes has been found to suppress androgen and gonadotropin activity [32]. An increase in testicular cholesterol can lower the concentration of androgens and consequently hamper spermatogenesis [99]. Chlorpyrifos can induce oxidative stress and cause oxidative damage and other histopathological changes in the reproductive system. It probably converts itself into chlorpyrifos oxon and decreases the level of serum testosterone and sperm count [100]. Chlorpyrifos has also been found to inhibit acetylcholine esterase activity in the brain resulting in interference of neuronal transmission involved in the synthesis and/or release of LH and FSH [101]. Suppressed production and low circulating levels of LH may subsequently disrupt the HPG axis, thereby blocking the testosterone synthesis pathway [102]. It also interferes with local regulators of testicular functioning, which is another factor behind reducing serum testosterone levels [103]. Furthermore, exposure to chlorpyrifos may induce oxidative damage to the Leydig cells thus, hindering normal testosterone biosynthetic pathway [104]. Chlorpyrifos-induced oxidative stress may manifest the combined effect of excessive ROS accumulation and reduced activity of antioxidant enzymes SOD and catalase [105]. Exposure to chlorpyrifos, even at a lower dose, may enhance testicular lipid peroxidation and diene conjugates while reducing the activities of SOD, catalase, and glutathione peroxidase. It also decreases the activity of steroidogenic enzymes 3β-HSD and 17β-hydroxysteroid dehydrogenase (17β-HSD), along with a decline in lipid–protein content of the testis [106,107]. Lowering testosterone concentration, in turn, impacts the negative feedback regulation of FSH, further hindering the process of spermatogenesis [108]. Chlorpyrifos has been found to inhibit the action of acetylcholinesterase, which results in the accumulation of acetylcholine neurotransmitters. This, in turn, suppresses the action of GnRH, which ultimately leads to testosterone deficiency. Over-accumulation of acetylcholine and decrease in testosterone levels contribute to the causes of ED [109].

A 12 week-long daily oral treatment of cypermethrin at 12.5 mg/kg body weight has resulted in decreased testosterone levels and testicular weight in the rat model [110]. When cypermethrin accumulates in the testes, oxidative stress is increased, and as a result, there is decreased viability of sperm along with damage to other testicular tissues [111]. Such organophosphorus compounds can also decrease serum testosterone levels by either directly inhibiting its production in Leydig cells or by increasing testosterone catabolism [112,113]. The decline in serum testosterone levels upon exposure to cypermethrin is possibly due to oxidative stress-mediated damage to testicular tissues, including Leydig cells. Oxidative stress reduces the viability of different cell types in testicular tissues, thus hampering testosterone synthesis [111]. Similar to organophosphorus pesticides, as mentioned above, cypermethrin has been found to decrease blood LH and FSH levels resulting in a reduction of testosterone concentration. This is suggestive of the fact that apart from testicular tissues, cypermethrin also disrupts the HPG axis [111]. Thus, cypermethrin-induced oxidative stress appears to be a manifestation of increased testicular lipid peroxidation, which leads to membrane degeneration and free radical formation. The free radicals, in turn, impair the antioxidant defense of enzymes, such as glutathione reductase, catalase and SOD [114]. Furthermore, cypermethrin mediates oxidative damage by reducing the activities of SOD, catalase, and glutathione peroxidase, which is characterized by increased malondialdehyde (MDA) levels in the testis [115]. It also has an anti-androgenic effect, which is mediated by the non-classical testosterone-signaling pathway involving mitogen-activated protein kinase (MAPK). Src kinase is a signaling protein of this pathway, and it interacts with androgen receptors on the Sertoli cell to elicit androgen response. Cypermethrin suppresses the interaction of androgen receptor and Src kinase, which interferes with gene expression in testosterone-mediated MAPK signaling pathway in Sertoli cells [116]. As cypermethrin has been reported to interfere with the synthesis, secretion, transport, binding, action, or elimination of hormones [117], this pesticide poses a risk of ED from the hormonal perspective. It may also be hypothesized that cypermethrin can induce ED by disruption of NOS activity mediated by increased oxidative stress.

**Figure 2:**
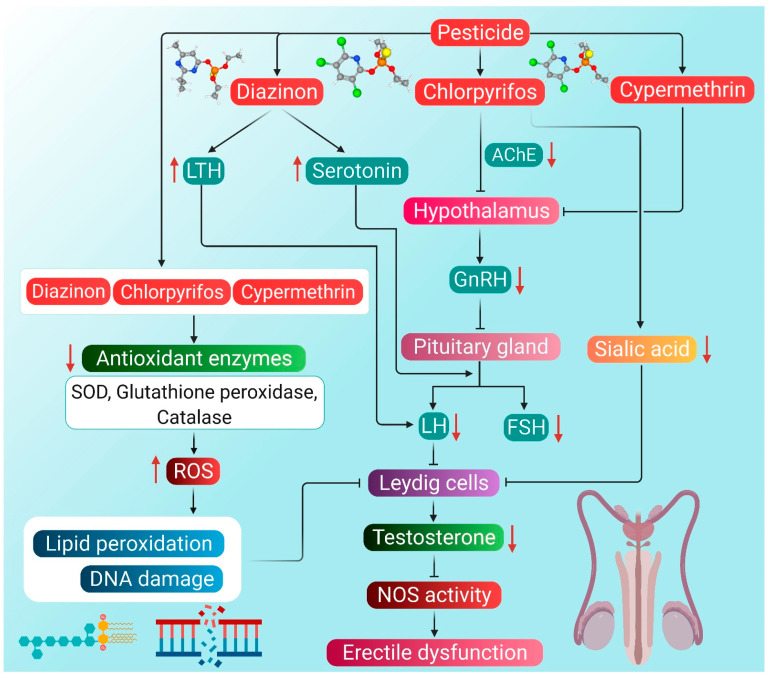
Probable mechanism of association of pesticide-induced ROS with hypogonadism and ED. Diazinon enhances LTH levels, which in turn lowers the activity of LH. Diazinon also stimulates producing serotonin that inhibits the action of both LH and FSH, thereby decreasing testosterone levels. Chlorpyrifos can reduce the activity of AChE in the hypothalamus. Consequently, the entire HPG axis is disrupted, leading to reduced testosterone production. Chlorpyrifos also reduces the concentration of sialic acid, which then inhibits Leydig cells. Cypermethrin disrupts the HPG axis by inhibiting the activity of the hypothalamus. Organophosphorus pesticides diazinon and chlorpyrifos, as well as pyrethroid pesticide cypermethrin, cause a decline in the antioxidant enzyme activity, thereby enhancing ROS generation. ROS causes lipid peroxidation and DNA damage, which inhibits the activity of Leydig cells, followed by reduced testosterone synthesis. Reduction in testosterone levels brings about the inactivity of the NOS enzyme, which ultimately leads to ED. Red arrows represent the increase and decrease of the respective substances, which have a negative impact on testosterone production, leading to hypogonadism and ED. (LTH: luteotropic hormone, AChE: acetylcholinesterase, GnRH: gonadotropin-releasing hormone, LH: luteinizing hormone, FSH: follicle-stimulating hormone, SOD: superoxide dismutase, NOS: nitric oxide synthase, ED: erectile dysfunction, ROS: reactive oxygen species).

The potential mechanisms through which pesticides can induce hypogonadism and ED are presented in Figure 2.

### 5.2. Radiation

Radiations are everywhere, and everyone is surrounded by different kinds of radiation sources. People are often exposed to various radiation sources, including radar, laptop, cell phone, microwave oven, Wi-Fi, television and radio transmission, medical equipment like X-ray machines, and radiotherapy [118,119,120,121,122]. A short-term 4 h exposure to 2.4 GHz of radiation causes a progressive decrease in sperm motility and a significant increase in sperm DNA fragmentation in men [118]. Whereas prolonged exposure to radiation emitted from 4G smartphones can diminish the reproductive potential, as demonstrated by a rat study [123]. Radiation is mainly classified into non-ionizing and ionizing, although nonionizing radiation is of two types—electromagnetic fields (EMF) and radio frequency (RF) [122]. Occupational exposure or even living nearby radar sites can cause marked testicular damage [122]. A study on prostate cancer patients undergoing external beam radiation therapy (EBRT) revealed a risk of permanent and persistent testosterone deficiency with elevated levels of LH and FSH that can ultimately lead to hypogonadism [124]. A common side effect of prostate cancer radiation or radiotherapy is ED [125]. Radiotherapy has been found to be more deleterious than chemotherapy for testicular cancer patients [126,127,128]. The radiotherapy doses (from 3000 to 7000 cGy) applied to treat cancer patients have been found to exert embryotoxic, mutagenic and teratogenic effects [129,130]. This included decreased sperm count and motility and increased chromosomal abnormalities in cancer patients after irradiation [131,132].

#### 5.2.1. Nonionizing Radiation

The networking of radiofrequency electromagnetic radiation (RF EMR) in the environment could be defined by the term “electro-pollution” or “electro-smog”, which has been listed with other environmental pollutants such as air, water, soil, and noise pollution [133]. Nonionizing radiation is emitted mainly from cell phones, cell phone towers, wireless devices, microwave ovens, radars, etc. [123]. Radiation emitted by smartphones has a negative impact on reproductive health [134,135]. Keeping cell phones near genitals or within 50 cm from genitals is associated with Leydig cell damage and reduction in testosterone levels along with abnormalities in sperm parameters [136]. Leydig cells play a vital role in testicular function. Therefore, any damage may hamper spermatogenesis and eventually cause infertility [137]. Additionally, electromagnetic radiations (waves of EM field) can induce changes in the motility parameters of spermatozoa [138,139,140].

Exposure to RF EMR from sources, such as 2.45 GHz Wi-Fi transmittermay cause a decline in the testosterone level [141,142,143,144]. Lin et al. [145] also investigated the effects of 1950 MHz radiation exposure on mice and reported the inhibition of testosterone production by Leydig cells. While exerting such effects, radiation may first disturb the Ca^2+^/CaMKI turnover in Leydig cells and thereafter inactivate the RORα clock gene. Radiation further downregulates its target genes *StAr*, P450 side-chain cleavage (*P450scc*), *3βHSD*, *P450c17*, and is involved in testosterone synthesis [146]. Exposure to 900 MHz radiofrequency for 10 days has been found to decrease the level of testosterone in rodent models [147]. Testosterone plays an important role in maintaining spermatogenesis and the physiological functions of seminiferous tubules. Hence, the decline in testosterone level could possibly hamper the process of spermatogenesis [148,149].

Exposure to Wistar rats with 900 MHz or 0.9 W/kg cell phone radiation was investigated to decrease the levels of histone kinase, SOD, and glutathione peroxidase activity along with increased catalase and MDA. The fluctuations were detected due to overproduction in radiation-induced ROS levels, which clearly indicates infertility in male rats after the exposure [24]. Kesari et al. also suggested an adverse impact of cell phone radiation on semen quality, particularly a decline in sperm count, motility, viability, morphology and increase in apoptosis [24]. In another study, when rats were exposed to a 10 GHz frequency of XeThru X4 radar for 90 days, the levels of serum testosterone and sex-hormone-binding protein have been found to decrease [150]. Another animal study after the exposure to 2.45 GHz showed an alteration in testicular histoarchitecture, decreased seminiferous tubule diameter, sperm count, sperm viability, and serum testosterone level, along with increased total ROS, NO, MDA levels. The expression of p53, Bax, active-caspase-3 in testes was also upregulated, while the expression of Bcl-xL, Bcl-2, procaspase-3, and PARP-1 was reported to be downregulated [151].

Exposure to RF EMFs originating from gadgets like microwave ovens and cell phones could generate seminal ROS leading to oxidative stress [134,152]. The generation of excessive superoxide may decrease glutathione peroxidase activity, while increased levels of hydrogen peroxide (H_2_O_2_) stimulates catalase activity [153]. Moreover, increased MDA level was noted due to an imbalance of charge in unsaturated fatty acids, which also triggers free radical production [154]. Exposure to microwave radiation causes apoptosis via the p53-dependent Bax-caspase-3-mediated pathway [151]. An increase in ROS and generation of NO starts producing free radicals, which can cause severe testicular oxidative damage in the form of lipid peroxidation and the formation of carbonyls [155]. Increased levels of ROS and NO change the redox status of testis and may also activate p53 for the trans-regulation of cell survival/death determining proteins [156]. Such an increase in oxidative stress may cause DNA strand breakage that may upregulate p53 expression [151]. An increase in ROS may also induce apoptosis by regulating the phosphorylation and ubiquitination of Bcl-2 family proteins, which may result in an increase of pro-apoptotic protein levels and a decrease of antiapoptotic protein expression. ROS increases Bax expression and suppresses Bcl-2 expression [157]. It can cause oxidation in the mitochondrial pore of sperm and disrupt mitochondrial membrane potential, too, which may further result in the release of cytochrome C. Such an increase in the level of cytochrome C leads to forming of apoptosomes and activation of caspase cascades [158,159]. Increased Bax/Bcl-2 ratio and cytochrome C level causes proteolytic cleavage of the initiator caspase procaspase-3 into an effector caspase active caspase-3. The active caspase-3 may cleave its substrate nuclear protein/DNA repair enzyme poly-(ADP) ribose polymerase-1 (PARP-1). This, in turn, may cause apoptosis of somatic cells as well as germ cells in the testis. Thus, production of spermatozoa may be delayed, and sperm count may decline, ultimately leading to infertility [151,160].

#### 5.2.2. Ionizing Radiation

Radiation, such as X-ray, α-ray, β-ray, has been characterized and listed under ionizing radiation. Ionizing radiations are much more deleterious than nonionizing radiations [123]. Adverse effects of such radiation on spermatogenesis are mediated through the testes and exhibit further detrimental consequences on androgen production [124,161]. Nicholas et al. [162] exposed their patients to photon-based radiotherapy (RT) and reported decreased serum testosterone levels. Another study on the exposure of patients with low prostate cancer risk to 76 Gy intensity-modulated radiotherapy (IMRT) for 36 months showed a decline in their testosterone levels [163]. Similarly, patients receiving EBRT also recorded a fall in their testosterone levels [164]. Pompe et al. [165] conducted a study on men who underwent EBRT for 2 years and revealed that 75% of patients underwent a significant decline in testosterone levels with up to 40% increase in the rates of biochemical hypogonadism. They further suggested that age might play a major role in the decline of testosterone levels in older men. In a rat study, Filchenkov et al. [166] reported that the exposure to acute external γ irradiation (0.5 Gy from 137Cs source, 10.33 × 10^−4^ Gy/s) for 90 days causes fluctuations in testosterone levels. In addition, a sharp upsurge in testosterone level occurred post-exposure to ionizing radiation in venous blood accompanied by decreased testosterone-binding globulin (TeBG), indicating the inhibition of the hormone in the tissues.

The EBRT of clinically localized prostate cancer patients for 3 months was found to reduce the serum testosterone levels from a pretreatment range of 185–783 ng/dL (with mean and median of 400 and 390 ng/dL, respectively) and a post-treatment range of 163–796 ng/dL (mean and median of 356 and 327 ng/dL, respectively) [167]. The decline in post-EBRT testosterone level could be linked with the radiation accumulated in the testes. Although, the testes are very sensitive to radiation, and spermatogenesis can be suppressed by radiation, even at doses as low as 30 cGy [168]. Testicular exposure to radiation of about 200 cGy (which is less than that given in EBRT) has resulted in Leydig cell damage, as evident from enhanced LH levels and lowered testosterone levels [167]. With increasing age, the sensitivity of Leydig cells towards radiation may increase [161,169]. In a rat study, when a group of animals was exposed to 7.5 Gy radiation daily for 5 days in the lower pelvis, decreased intracavernous pressure (ICP) in the penis was observed, which is a direct measure of ED [170]. In another study, rats were exposed to 20 Gy radiation, and ICP was measured for 2, 4 and 9 weeks. This revealed a time-dependent decrease in ICP. Oxidative DNA damage was increased in corpora cavernosa and prostate, whereas elevated lipid peroxidation was noted in corpora cavernosa [171]. Ji et al. [172] also noted that radiation-induced oxidative stress may damage the DNA. In their study, rat testes were irradiated with a single dose of 4 Gy X-ray that reduced the sperm count, motility, and testicular weight. It also caused a reduction in serum testosterone level and a distortion in the architecture of the seminiferous tubules. Ezz and coworkers irradiated rats with γ radiation, which induced oxidative stress along with a sharp rise in testicular MDA levels. It also reduced the SOD activity and serum testosterone levels [173]. Moreover, when mice were irradiated with 0.258 Gy X-ray twice a day for 4 days a week, it resulted in elevated ROS levels, lipid peroxidation, serum lactate dehydrogenase (LDH) activity, along with a reduction in testicular glutathione concentration. Apart from that, increased activities of antioxidant enzymes, such as glutathione reductase, glutathione peroxidase, catalase, superoxide dismutase and glutathione-S-transferase were also observed in the testes of irradiated mice. Sperm count, motility and levels of testosterone were also decreased. Increase in serum LDH activity indicates cellular damage as the LDH is considered as a diagnostic marker of cellular damage [174].

Ionizing radiation can cause accumulation of collagen in the penile tissues, loss of smooth muscle and even induce fibrosis [170] and downregulate steroidogenic and spermatogenic activities [173]. Radiation in the prostate causes an upregulation of NADPH oxidase subunits gp91phox and Nox4 that result in forming ROS in the penile shaft and oxidative stress due to the imbalance between ROS and Nrf2 protein [171,175]. Testicular tissues are vulnerable to oxidative stress-induced pathologies because of the inherent abundance of highly unsaturated fatty acids, high metabolic activity, high mitotic activity, and the presence of potential ROS-generating systems [174]. ROS and oxidative stress are detrimental to the mitochondrial membranes, proteins, carbohydrates, RNA and DNA [176]. Increased levels of ROS have been associated with lower ICP/MAP ratio. Indeed, ROS such as superoxide (O_2_^−^) and H_2_O_2_ have been associated with long-term inflammation of tissues [177]. Superoxide reacts with NO in the penile tissues and results in ED [21,178]. Although, NO reacts with O_2_^−^ to form peroxynitrite [178], which then reacts with tyrosyl residues of protein to inactivate superoxide dismutase [179]. Superoxide dismutase is known to remove O_2_^−^ radicals from the body [94], and when it is inactivated, the amount of O_2_^−^ increases in the cell [179]. This event further reduces the concentration of available NO and generates peroxynitrite. Peroxynitrite, in turn, mediates the relaxation of cavernosal smooth muscles, which includes generating cGMP that causes a powerful and strong guanylyl cyclase inhibitor oxadiazoloquinoxalin-1-one (ODO) to prevent the relaxation of cavernosal smooth muscle [180]. This gives rise to ED because erection occurs as a result of relaxation of cavernosal smooth muscles [181].

**Figure 3:**
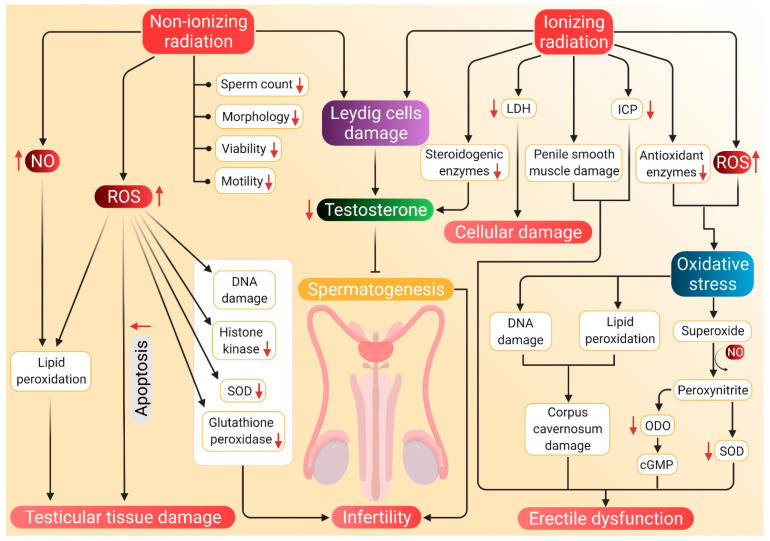
Probable mechanism of association of radiation-induced ROS with hypogonadism and ED. Nonionizing radiations damage the Leydig cells, which reduces testosterone synthesis and subsequent inhibition of spermatozoa. Such radiations negatively affect sperm parameters, including count, motility, morphology, viability and motility. Nonionizing radiations also stimulate NO activity, which along with ROS, brings about lipid peroxidation and damage to testicular tissues. These tissues are also damaged by apoptosis mediated by elevated ROS levels. Nonionizing radiation-induced ROS further causes DNA damage and decreases the concentration of histone kinase along with a reduction of SOD and glutathione peroxidase activity. Whereas ionizing radiations cause a decline in testosterone production by damaging Leydig cells and by inhibiting the activity of steroidogenic enzymes. Such radiations enhance LDH activity, which results in cellular damage. Damage to the penile smooth muscles and reduced ICP may cause ED. ED is also caused by decreased cGMP levels and damage to the corpus cavernosum, which are brought about by ionizing radiation-induced oxidative stress. Red arrows represent the increase and decrease of the respective substances and sperm parameters beyond harmful levels. (NO: nitric oxide, SOD: superoxide dismutase, LDH: lactate dehydrogenase, ICP: intracavernosal pressure, ODO: oxadiazoloquinoxalin-1-one, cGMP: cyclic guanosine monophosphate, ED: erectile dysfunction, ROS: reactive oxygen species).

The potential mechanisms through which radiation can induce hypogonadism and ED are presented in Figure 3.

### 5.3. Air Pollution

Humans are exposed to air pollution from different sources, such as refineries, power stations, municipal incineration, automobiles, cars, railways, jets, several other industries, forest fires or agricultural burnings, volcanic eruptions, etc. Substances like heavy air pollutants, particularly the metals cadmium (Cd) and lead (Pb), particulate matter (PM), sulfur dioxide (SO_2_), volatile organic compounds, polycyclic aromatic hydrocarbons, carbon monoxide (CO), and ozone (O_3_) pollute the air and cause various diseases [182].

#### 5.3.1. Heavy Metal Pollution

Heavy air pollutants, such as Cd, most often are adsorbed in PM2.5, which is a mixture of minute particles and water droplets in the air and imparts toxic effects on the reproductive tract [183]. Cadmium accumulation in testis generates high levels of ROS, subsequently surpassing the antioxidant capacity of testes, which leads to lipid peroxidation, degeneration of seminiferous tubules, testicular hemorrhage, testicular necrosis, abnormalities in Leydig cells, fibrosis and reduced testicular size [26].

Testosterone is prone to direct Cd²^+^ exposure. A rat study showed that exposure to CdCl_2_ decreased the serum testosterone level, testicular 3β-HSD and 17β-HSD activities, whereas these are key enzymes for testosterone biosynthesis [184]. CdCl_2_ toxicity also induces significant downregulation in the mRNA levels of cytochrome 450 cholesterol side-chain cleavage enzyme, androgen receptor and steroidogenic acute regulatory protein [185]. Moreover, Cd inhibits the production of testosterone in Leydig cells by downregulating the expression of dihydrolipoamide dehydrogenase (DLD) and decreasing the levels of intercellular cyclic adenosine monophosphate (cAMP) [186]. Kresovich et al. [187] noted that men with higher Pb exposures have increased levels of albumin-bound testosterone. Moreover, among smokers, the level of testosterone and concentration of blood Pb showed a positive trend. When rats were supplied with 50 mg/L lead acetate (PbAc) in drinking water, it resulted in a significant decrease in testosterone levels and E2 in serum as well as testis. Pb also significantly downregulates the expression and mRNA level of *Cyp19* (P450 arom) that may be due to activation of protein kinase C. Decline in the level of serum testosterone may be due to Pb-induced damage to Leydig cells. Damaged cells can still manage to produce testosterone; however, it fails to reach circulation [188]. Disruption in E2 can cause male infertility as it plays an important role in regulating spermatogenesis by exerting its function upon binding with specific estrogen receptors (ERα, ERβ) along with maturation and motility of sperm [189,190,191].

A study by Elmallah et al. showed that injecting rats with 6.5 mg/kg of CdCl_2_ intraperitoneally for 5 days elevated Cd concentration in testicular tissues, decreased testicular weight and testosterone level, with increased lipid peroxidation (as indicated by MDA and NO). Additionally, activities of enzymes, such as SOD, catalase, glutathione peroxidase and glutathione reductase, were diminished. Upregulation of proapoptotic proteins, Bcl-2-associated-X-protein (*BAX*), and *TNF*-α were detected, whereas the antiapoptotic, B-cell lymphoma 2 (*BCL2*) gene was downregulated. In addition, an increased level of TNF-α along with a decreased number of proliferating cell nuclear antigens (PCNA) was detected [192]. In another study, rats receiving a daily dose of 4.28 mg/kg CdCl_2_ (2.62 mg/kg Cd/day) for 7 days showed damage in the epithelium of seminiferous tubules, decreased concentration of serum testosterone and SOD activity [193]. Similarly, when rats were given a single oral supplementation of 10 mg/kg body weight of CdCl_2_, a significant decrease in the level of serum testosterone and increased lipid peroxidation (MDA) were noted. Increased level of MDA suggests that CdCl_2_-induced oxidative stress causes testicular damage by increasing ROS production [194]. When 2.5 mg/kg body weight CdCl_2_ was orally supplemented to two groups of male rats for 14 and 42 days, respectively, it caused a duration-dependent decrease in testosterone levels, FSH, and LH along with decreased semen quality parameters and gonadosomatic index [195]. Cadmium accumulation in testis generates high levels of ROS, subsequently surpassing the antioxidant capacity of testes. This leads to lipid peroxidation, degeneration of seminiferous tubules, testicular hemorrhage, testicular necrosis, abnormal Leydig cells, fibrosis and reduced testicular size [26]. Lipid peroxidation in mitochondria may disintegrate the ultrastructure of a mitochondrial membrane that may affect membrane-bound lactate dehydrogenase, and LDH function, resulting in its inhibition. The activity of the LDH enzyme is closely associated with spermatogenesis and male testicular development. A decrease in the activity of this enzyme represents a defect in spermatogenesis [196]. Oxidative stress also depletes the DNA contents of dividing spermatogenic cells [192]. Exposure to Cd may increase the concentration of testicular NO by the induction of NOS following increased TNF-α level, which could be associated with IL-4 reduction. Endothelial NOS expression increases in germ cells exposed to Cd along with higher apoptotic indices that showed the inhibitory effect of NO on spermatogenesis. Apart from increased NO, reduced activity of SOD, catalase, and consequent oxidative stress may damage testicular tissues [193]. Cd^2+^ structurally resemble Ca^2+^ [197] in the cell membrane that may change the integrity of the membrane of sperm acrosome, which can lead to abnormal acrosomal reaction and hamper fertility. Cadmium can modulate the commencement and interval of acrosome reaction and incidence of a separated flagellum [198]. A decrease in the antioxidant enzyme activity may be either due to the inhibition of enzyme activity by Cd or Cd-induced transcription of the corresponding genes [192]. Oxidative stress-induced sperm deficiency is a major idiopathic factor of male infertility. Sperm and testicular Leydig cell mitochondria are highly susceptible to Cd-induced oxidative stress. Cadmium may cause spermatotoxicity by either affecting spermatogenesis via oxidative stress or disrupting the HPG axis [195,199].

A study by Kelainy and coworkers demonstrated that oral supplementation of 20 mg/kg PbAc to rats for 10 days increases the levels of ROS in testicular tissues. PbAc also increased the levels of lipid peroxidation and decreased the catalase activity and total antioxidant capacity. A decrease in the levels of serum testosterone, FSH and LH were also detected. Enhanced levels of lysosomal enzymes ACP, ß-NAG, and β-GAL in testes were also observed where the accumulation of Pb in testicular tissues was also reported [200]. More recently, Dorostghoal et al. [201] noted a reduction in testicular weight, the diameter of seminiferous tubules, epididymal sperm count, serum testosterone, and testicular levels of SOD and glutathione peroxidase in rats consuming drinking water containing 0.1% PbAc for 70 days. In another study, 50 mg/kg body weight Pb when orally supplemented to rats for 4 weeks resulted in a reduction of glutathione, catalase, and SOD activity along with a decline in GnRH and testosterone levels [202]. Interestingly, an in vitro study involving rat Leydig cell line R2C reported decreased progesterone (the precursor of testosterone) release along with a decrease in the expression level of StAR, CYP11A1 and 3β-HSD proteins upon incubation with Pb (50, 100, 200, 400 μM) for 24 h [203]. Lead affects male reproductive function either directly by hampering spermatogenesis and sperm function or indirectly by disturbing the HPG axis [201,204,205]. Moreover, exposure to Pb causes lipid peroxidation and infertility in men by suppressing the creatine kinase activity of sperm, which in turn hinders normal sperm metabolism [206,207]. It can inactivate endogenous antioxidants that cause an imbalance in antioxidant/prooxidant status resulting in oxidative stress. Imbalance in antioxidant enzyme activities and decrease in GnRH and testosterone levels is also known to cause male infertility [202]. Lead absorbed in blood and tissues produces ROS, such as O_2_^•−^, H_2_ O_2_, OH^•^ and lipid peroxides [208]. Over-production of ROS causes oxidative stress by overwhelming the body’s antioxidant defense [201,209]. ROS can oxidize structural proteins of blood and tissues and can also inhibit the proteolytic enzyme [210]. Oxidation of proteins causes fragmentation of amino acids that change the structure of proteins and the function of enzymes. ROS also induces genetic transcription, protein transformation and can change the lysosomal system and the proteasomes—the two major pathways by which proteins are degraded [211]. Lead-induced oxidative stress can change the expression of antioxidant enzymes, such as superoxide dismutase 2 (SOD2) and galactoside acetyltransferase (GAT) that, in turn, decreases progesterone production [203]. PbAc can also decrease DNA synthesis by suppressing DNA polymerase B [212].

#### 5.3.2. Particulate Matter (PM2.5) Pollution

PM2.5 is a complex mixture of extremely small particles and liquid droplets that get mixed easily into the air [213]. PM2.5 pollution affects different organ systems, including the reproductive system [182]. It is also one of the probable reasons for ED [83,214]. These may cause damage to the arteries that supply blood to the penis and reduce the blood flow in the penis [215]. In a rat study, three groups of animals, when exposed to 0.8, 1.6 and 3.6 mg of PM2.5 once a week for 6 weeks, the ratio of ICP to mean atrial pressure (MAP) (i.e., ICP/MAP ratio) decreased, and the decline was higher in the high dose group. The ratio of smooth muscles to collagen was also reduced, and producing ROS was found to increase [25].

In an experimental model of male C57 black 6 (C57Bl/6) mice using a whole-body exposure system that mimics real-world exposure to air pollution for four months, exposure to concentrated ambient PM2.5 (CAP) or filtered air (FA) was found to affect sperm count, major hormones of the HPG axis, testicular histology, and mRNA expression of testosterone biosynthesis genes [216]. The negative effect of CAP exposure on sperm count, testicular germ cells, circulating FSH and testosterone levels, hypothalamic GnRH mRNA levels are strongly suggestive of the harmful impact of ambient PM2.5 on the male reproductive system [217]. PM2.5 exposure adversely affects the hypothalamic–pituitary axis and testicular spermatogenesis, which can potentially cause sperm alterations [218]. It negatively influences the male reproductive system through suppression of the HPG axis [217]. Testosterone is one of the major components of the HPG axis [219], and the pituitary hormone LH is crucial for the expression of testosterone biosynthesis enzymes. CAP exposure remarkably decreases the testicular expression of P450scc, 17β-HSD, and StAR mRNA, which further decreases the LH level [217]. This indicates a negative correlation of testosterone level with PM2.5 [217]. A marked decrease in FSH level is corroborated by the decrease of the FSH target gene. Exposure to high concentrations of PM2.5 disturbs various stages of spermatogenesis, damages the basement membrane and tunica propria as well as reduces the number of germ cells [220].

The mechanism related to particulate matter pollution is still unclear, although the evidence presented in this study indicates the hypothesis that oxidative stress could be a responsible factor behind the harmful effects of PM2.5 on sperm parameters and fertilization. Several researchers reported that oxidative stress can lower producing endothelial NO that can increase the smooth muscle tone, induce vasodilation, and diminish the ability of the penis to achieve an erection [221,222]. Elevated exposure to PM2.5 increases ROS production in the body, which may enhance TNF-α level. This, in turn, can again increase the production of ROS. However, excess production of ROS may decrease the activity of endothelial NO synthase and lead to a reduction in the bioavailability of NO for the relaxation of smooth muscles of the cavernosa [25]. Oxidative damage caused by free radicals has been recognized as one of the important mechanisms for PM2.5 to induce biological activity. When cells are stimulated by oxidative damage signals, ROS are formed, and antioxidant substances are depleted, or their activity decreases, thus impacting the cellular balance. These cause excessive ROS to attack biological macromolecules inside the cells, such as lipids, proteins, and DNA. It may result in oxidative damage leading to DNA strand break, induction of apoptosis and even carcinogenesis [183]. ROS is an important factor that induces cell death and spermatogenic cell damage [223,224]. Seminal ROS can strike a wide range of essential biomolecules, such as proteins, lipids, carbohydrates, and nucleic acids, and affect their functions. Their impact may consequently be involved in DNA damage, decreased sperm motility, reduced sperm viability, sperm dysfunction, and semen hyperviscosity [225]. Lipid peroxidation of sperm plasma membranes by ROS causes reduced membrane fluidity [225,226]. Exposure to PM2.5 has also been linked to inflammation and oxidative stress [83] and hence may potentially cause ED of vascular origin through veno-occlusive or arteriogenic pathways [227]. PM2.5 exposure impairs erectile function, which is mediated via induction of local and systematic inflammatory responses, oxidative stress, hazardous effect on angiogenesis, and oxidative DNA damage [228]. It has been associated with atherosclerosis [229] and endothelial dysfunction [214], and both are strongly linked with ED.

**Figure 4:**
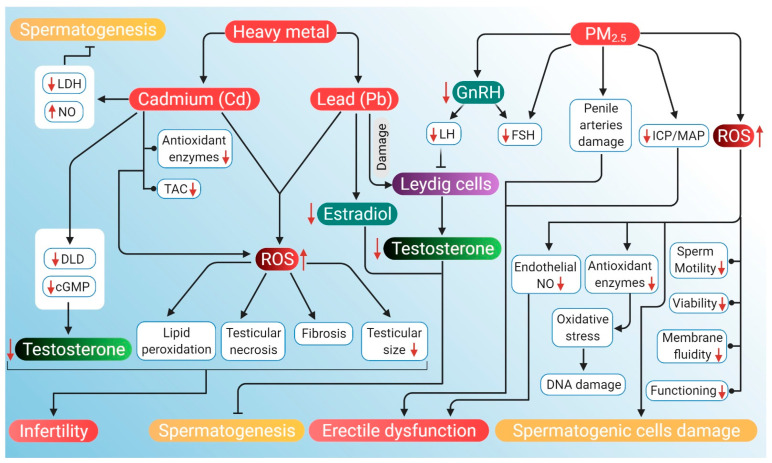
Probable mechanism of association of ROS induced by air pollutants (heavy metals Cd and Pb, and PM2.5) with hypogonadism and ED. Heavy air pollutant Cd increases NO and decreases LDH activity to inhibit spermatogenesis. It also lowers DLD and cAMP levels, which reduces testosterone. Cd decreases antioxidant enzymes along with a decrease in total antioxidant capacity. Through these mechanisms, Cd increases ROS production, which in turn causes lipid peroxidation, testicular necrosis, fibrosis and reduced testicular size. These factors, together with lowered levels of testosterone, contribute to the cause of infertility. Another heavy metal Pb reduces testosterone levels and estradiol, which leads to the inhibition of spermatogenesis. PM2.5 downregulates GnRH activity, thereby declining LH and FSH levels. Reduction in LH inhibits the action of Leydig cells, thereby lowering testosterone production. PM2.5 also induces ED by damaging penile arteries, reducing the ICP/MAP ratio and inhibiting endothelial NO activity. PM2.5 further stimulates the generation of ROS that can damage DNA and spermatogenic cells. PM2.5-induced ROS may also have a negative impact on sperm quality, particularly motility, viability, functioning and membrane fluidity. Red arrows represent the increase and decrease of the respective substances and sperm parameters beyond harmful levels. (NO: nitric oxide, LDH: lactate dehydrogenase, DLD: dihydrolipoamide dehydrogenase, cAMP: cyclic adenosine monophosphate, TAC: total antioxidant capacity, GnRH: gonadotropin-releasing hormone, LH: luteinizing hormone, FSH: follicle-stimulating hormone, ICP: intracavernosal pressure, MAP: mean arterial pressure, ED: erectile dysfunction ROS: reactive oxygen species).

The potential mechanisms through which air pollution can induce hypogonadism and ED are presented in Figure 4.

### 5.4. Other Endocrine-Disrupting Chemicals

In general, environmental endocrine-disrupting chemicals have anti-androgenic effects that are mediated by mechanisms, such as interference with the androgen receptor, androgen production, metabolism, or signaling in the HPG axis [230]. Apart from making our daily life easier, plastic-originated agents and other endocrine-disrupting chemicals cause harm not only to the environment but also to the fertility and health of men. Polycarbonate plastics and epoxy resins, made of polymers of bisphenol A (BPA), affect the male reproductive system. BPA also acts as an endocrine disruptor and causes testosterone deficiency, decreases sperm count and motility, along with the damage of normal morphology, DNA of sperm and the process of spermatogenesis [231]. Another common chemical used as a plasticizer is phthalate esters, which are known to cause testicular toxicity in rodent models apart from affecting spermatogenesis, Leydig cells and testosterone production [232]. Perfluoroalkyl substances (PFAS) are used in the manufacturing of polymers, personal care products and nonstick cookware, whereas polychlorinated biphenyls (PCB) are used as a coolant and lubricant in electronic equipment. These substances can also generate ROS leading to an oxidative stress-induced decline in testosterone and androgen production [233,234].

#### 5.4.1. Agents Originating in Plastics

Factory workers regularly exposed to high levels of BPA for four years have reported reduced sexual function and a six-fold increase in the risk of coitus frequency and ejaculatory dysfunctions. Such men have also exhibited an increased risk of decline in libido and failure in achieving penile erection [235]. Men who are exposed to BPA have a significantly higher likelihood of reduced sexual desire, ED, and ejaculatory dysfunction [236]. Workers in BPA and epoxy resin manufacturing companies have also been reported with lower sexual functions in terms of erectile function, orgasmic function, sexual desire, and overall satisfaction in sex life [235]. Previously, changes in endogenous sex hormone levels have been found in BPA-exposed men in terms of estrogen, androgen, and gonadotropin, as well as sex-hormone-binding globulin (SHBG) concentrations [237]. Elevated total urinary BPA concentrations and lower FSH levels have also been seen in the workers exposed to BPA diglycidyl ether (BADGE) from spraying epoxy resin [237]. BPA exposure during the perinatal and postnatal periods also affects the endocrine functions of the HPG axis. At the hypothalamic–pituitary level, BPA exposure results in the upregulation of *KiSS-1* expression (that encodes Kisspeptin protein), GnRH and FSH mRNA. At the gonadal level, BPA causes inhibition in the expression of testicular steroidogenic enzymes and the synthesis of testosterone [238]. Furthermore, BPA causes a reduction in sperm production, including compromising the integrity of the acrosome and plasma membrane as well as a reduction in mitochondrial activity. This, in turn, disrupts the HPG axis resulting in a state of hypogonadotropic hypogonadism. BPA adversely affects male sexual function through its estrogenic and antiandrogenic effects. BPA exposure reduces the synthesis of LH, which in turn negatively affects plasma and testicular testosterone levels. It can further reduce the expression of steroidogenic enzymes and cholesterol carrier protein in Leydig cells [239]. The expression of StAR protein has also been reported upon BPA exposure, which inhibits the normal regulatory pathway of steroidogenic enzymes. At high doses, expression of StAR and peripheral benzodiazepine receptor (PBR) involved in testosterone biosynthesis further decreases testosterone levels [239]. Exposure to BPA has been found to induce ROS generation, which leads to increased levels of lactoperoxidase and activation of antioxidant enzymes. It can also alter the levels of SOD and chloramphenicol acetyltransferase, indicating oxidative stress. Enhanced ROS levels and compromised antioxidant enzyme levels can impair spermatogenesis [240], reduce the number of spermatids, alter the epithelial height and seminiferous tubules, and decrease the concentration of testosterone [241]. When BPA enters the body, it can mimic the effects of estrogen and may also hinder the release of male sex hormones, including testosterone, which may be a probable reason for BPA-induced sexual dysfunction, including ED.

Men exposed to di-n-butyl phthalate (DBP) and di-2-ethylhexyl phthalate (DEHP) for almost a year in an unfoamed polyvinyl chloride flooring producing factory have reported higher levels of phthalates, such as mono-n-butyl phthalate (MBP) and mono-2-ethylhexyl phthalate (MEHP) in their urine and blood samples along with lower levels of free testosterone. In fact, MBP and MEHP levels in the factory workers have been 5–100 times higher than that in the unexposed group of men [232]. When dosed daily with gavage of DEHP for 30 days, adverse effects have been noted on testicular physiology and testosterone production in the rat model [242]. Moreover, administration of DEHP appears to induce histomorphological changes of rat testis, including deformed seminiferous tubules, aggregated chromatin, multiple vacuoles, swollen mitochondria, apoptotic germ cells and Sertoli cells, as well as increased Leydig cell numbers [242]. DEHP treatment to mouse Leydig cells TM3 for 24 h after pretreatment with vitamin C or U0126 (an inhibitor of methyl ethyl ketone) has been associated with disturbance in the HPG axis along with a reduction in serum testosterone, LH and FSH levels [242]. DEHP exposure also results in the disturbance of the HPG axis, leading to impaired testosterone biosynthesis [243]. It can downregulate testosterone levels by inducing 5α-2eductase 2 expression in the testis via activation and phosphorylation of the ERK pathway [242]. DEHP-induced inhibition of testosterone production in Leydig cells has been associated with a decline in pituitary LH secretion. Devitalization of pituitary LH secretion during development adversely affects the acquisition of steroidogenic capacity, thereby decreasing testosterone biosynthesis by Leydig cells [243]. The expression of genes associated with cholesterol synthesis, metabolism, transport, and storage in Leydig cells also decreases the testosterone level, which is associated with DEHP exposure [242]. After exposure to di (n-butyl) phthalate, mRNA expressions of proteins, such as scavenger receptor B 1 (SRB1) and StAR, have been found to be downregulated [244]. Detrimental effects of DEHP on Leydig cell steroidogenesis occurs through the modulation of testosterone-biosynthetic enzyme activity. DEHP causes decreased activity of the steroidogenic enzyme 17β-HSD, which reduces testosterone production in Leydig cells [243]. In general, phthalate compounds are capable of inducing oxidative stress in the male reproductive organs—mainly testis and epididymis. They impair the spermatogenic process by inducing oxidative stress and apoptosis in germ cells or target Sertoli cells, thus hampering spermatogenesis. Phthalates also impair the Leydig cell function by elevating ROS generation and decreasing the levels of steroidogenic enzymes [245]. MEHP can disrupt prepubertal Sertoli cell proliferation by increasing intracellular ROS levels [246]. 5α-reductases may play a significant role in the downregulation of testosterone levels following DEHP exposure. Phthalates are known to disrupt male reproductive development in an anti-androgenic fashion [247] and have been associated with a reduction in testosterone levels [232,248]. Low levels of testosterone have been associated with reduced sexual desire, decreased spontaneous erections, and ED [249,250,251]. However, the role of phthalate metabolites as potential risk factors of ED remains to be elucidated clearly.

PFAS are synthetic compounds that are suspected endocrine-disrupting chemicals having the ability to cause dysfunction to hormonally regulated body systems [252]. Widespread and regular daily exposure to PFAS occurs primarily through drinking water, diet, outdoor air, indoor dust, and soil [253]. A study involving 247 healthy young Danish men with a median age of 19 years has revealed a negative association of perfluorooctane sulfonate (PFOS), a type of PFAS with serum testosterone levels, but not with semen quality [254]. Based on testosterone levels and E2, a correlation of PFOS and perfluorooctanoic acid (PFOA) levels with the delay of puberty in children (boys and girls) has also been demonstrated [234]. Higher levels of PFOS exposure are significantly associated with decreased serum testosterone concentrations in the male, which is age-specific and stronger in older men [230]. CYP11A1, a protein-coding gene, catalyzes the conversion of cholesterol to pregnenolone, and steroidogenesis starts with this reaction in all mammalian tissues [255]. PFOA and PFOS probably target this regulatory pathway and may disrupt androgen production by the downregulation of CYP11A1 and CYP17A1 production [256,257]. In addition, PFOA or PFOS have a cytotoxic effect on Leydig cells, which are primarily related to androgen biosynthesis [256,257,258]. The negative correlation of PFOS and PFOA with total testosterone levels in men may be attributed to the vulnerability of Leydig cells towards PFOS and PFOA. In patients with high PFOS-PFOA levels, there is a tendency towards lower testosterone level, free androgen index (FAI) and inhibin-B with no alterations in LH and FSH levels [254]. Moreover, PFAS can activate peroxisome proliferator-activated receptor alpha and induce peroxisome proliferation. A possible mechanism of action for PFAS is generating oxidative stress, which severely damages DNA [259]. PFAS-induced ROS generation, when exceeds that of cellular antioxidant capacity, negatively influences male reproductive functions affecting the HPG axis either directly or indirectly. Excessive ROS production may also lead to apoptotic or necrotic cell death [260]. Furthermore, PFAS can indirectly cause enhancing circulating cortisol levels, leading to oxidative stress and reduced circulating testosterone levels [15]. However, the exact impact of PFAS exposure on ED is yet to be understood completely.

#### 5.4.2. Polychlorinated Biphenyl (PCB)

**Figure 5:**
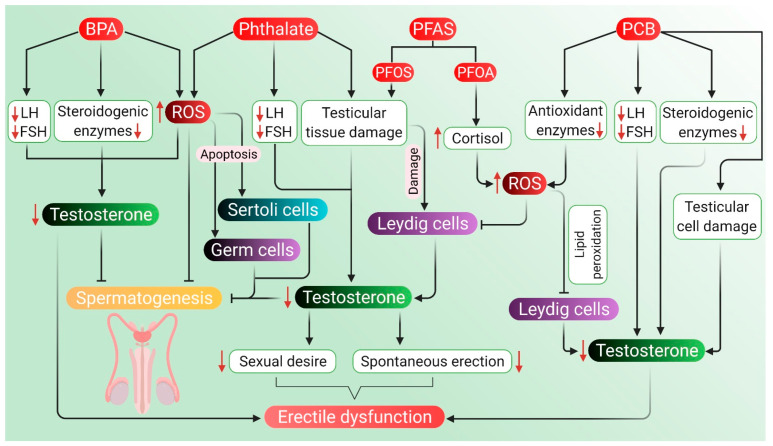
Probable mechanism of association of ROS induced by BPA, phthalates, PFAS and PCB with hypogonadism and ED. BPA downregulates the production of LH, FSH and steroidogenic enzymes along with upregulation of ROS, which, in turn, causes a decline in testosterone levels followed by inhibition of spermatogenesis. BPA-mediated ROS generation may also suppress testosterone activity and spermatogenic processes directly. Phthalates cause damage to testicular tissues and lower the levels of LH and FSH, which further reduces testosterone levels. ROS generation by phthalates may bring about the apoptosis of Sertoli cells and germ cells, ultimately inhibiting spermatogenesis. Lowered testosterone levels also result in diminished sexual desire and reduce spontaneous erection, which may lead to ED. PFAS, such as PFOS and PFOA, may also cause damage to Leydig cells leading to reduced testosterone levels. PFOS and PFOA may induce cortisol production, which leads to ROS generation, ultimately resulting in suppression of the hormonal activity of Leydig cells. PCBs damage testicular tissues and lower the activity of LH and FSH together with reducing the steroidogenic enzymes, which may lead to lowered testosterone levels. PCBs can also reduce the levels of antioxidant enzymes, and resulting in oxidative stress induces lipid peroxidation in Leydig cells, thus lowering the level of testosterone. PCB-induced reduction of testosterone may also give rise to ED. Red arrows represent the increase and decrease of the respective substances, which negatively impact testosterone level, thereby inhibiting spermatogenesis and causing hypogonadism and ED. (BPA: bisphenol A, PFAS: perfluoroalkyl substances, PFOS: perfluorooctanesulfonic acid, PFOA: perfluorooctanoic acid, PCB: polychlorinated biphenyls, LH: luteinizing hormone, FSH: follicle-stimulating hormone, ED: erectile dysfunction, ROS: reactive oxygen species).

The major route of exposure to PCB is through the ingestion of contaminated food, particularly fish. The lipophilic nature of PCBs helps these chemicals to degrade very slowly, enabling their bioaccumulation within the food chain [261]. Exposure to PCB153 has been reported to decrease serum testosterone levels [233]. To study the effect of PCB153 on Leydig cell and testosterone level, adult Leydig cells were exposed to different concentrations (10^−10^ to 10^−7^ M) of PCB for 6 and 12 h under basal and LH-stimulated conditions. The results indicated that PCB (10^−8^ and 10^−7^ M) treatments significantly inhibit basal and LH-stimulated testosterone production. In addition, the activity of steroidogenic enzymes, such as P450scc, 3-HSD and 17-HSD; enzymatic antioxidants, such as SOD, catalase, glutathione peroxidase, glutathione reductase, -glutamyl transpeptidase (-GT), and glutathione-S-transferase (GST); and nonenzymatic antioxidants, such as vitamins C and E were diminished in a dose- and time-dependent manner [262]. PCB exposure prevents the steroidal binding of testosterone and subsequently affects the bioavailability of free testosterone. First, steroidogenic enzyme activity within Leydig cells is reduced, ultimately leading to decreased biosynthesis of androgens. Second, there is decreased pituitary LH secretion secondary to disruption of the HPG axis. This, in turn, may lead to a condition of hypogonadism [263]. PCB153 exposure significantly decreases LH concentration, which indirectly impairs testosterone production via decreased stimulation of testicular Leydig cells [233]. Moreover, PCB also reduces pituitary response to low androgen levels [264]. These changes are accompanied by several alterations in the pituitary–testicular hormone axis. Plasma concentration of testosterone and dihydrotestosterone decreases because of decreased synthesis. As a result, the plasma concentration of LH fails to rise in response to the decreased testosterone levels, implying that PCB also disrupts a pituitary feedback mechanism [264]. Altered testosterone biosynthesis is also associated with reduced LH receptor-binding activity, decreased activity of steroidogenic enzymes as well as both enzymatic and nonenzymatic antioxidants [262]. PCB-induced reduction in Leydig cell receptor-binding activity has been associated with reduced activity of steroidogenic enzymes, such as P450scce, 3- and 17-HSDs and antioxidant enzyme activities in Leydig cells. These changes may be due to the elevated levels of ROS and lactoperoxidase [262]. Oxidative stress is believed to result in reduced levels of key enzymatic and nonenzymatic antioxidants in Leydig cells. This may cause a decline in testosterone secretion [265,266]. Therefore, the endocrine-disrupting properties of PCBs can modulate the normal functioning of endogenous hormones as agonists, as antagonists, or as mixed agonists-antagonists, particularly concerning estrogen or testosterone activity [267,268]. Moreover, male erection is controlled by a complex interplay of neural, vascular and hormonal factors [269,270,271]. Hence it is plausible that PCBs along with other industrial chemicals can directly or indirectly interfere with the action of sex hormones, such as androgens, thus negatively impacting erectile function [272]. The potential mechanisms through which plastic-originated agents and other endocrine-disrupting chemicals can induce hypogonadism and ED are presented in Figure 5.

Table 1 summarizes the effects of the aforementioned environmental factors on hypogonadism and ED. The exposure parameters and the respective findings has also been summed up in the table.

## 6. Future Perspectives

Environmental factors, such as organophosphorus pesticides (chlorpyrifos, diazinon, and cypermethrin), radiations (from EBRT and cell phones), air pollutants (mainly Cd and Pb), plastic-originated agents (particularly BPA) and other endocrine-disrupting chemicals can alter the oxidative balance, due to which spermatozoa may lose viability, motility, ionic balance and the proteome of sperm also undergoes changes [273]. The clinicians can monitor the mental health of patients suffering from LOH and adopt necessary therapeutic measures when required, which is rather complicated [71,274]. It is advised that men in the age range of 55–69 years with hypogonadism should be screened, monitored, and may also be considered for testosterone-replacement therapy (TRT) [275]. Testosterone trials showed that treating such men with TRT can lead to moderate improvement in sexual function and libido, level of hemoglobin, the mineral density of bone, lean body mass, physical strength, short-term endothelial functions, including arterial stiffness and vasodilatory response, and a slight positive effect on the mood [71,276,277]. However, the clinician may first try to treat the modifiable risk factor through exercise, weight loss and then should consider these important issues relating to environmental pollutants while treating men with hypogonadism and ED [3,274]. TRT is an evidence-based method that is prescribed, and, moreover, it can normalize the testosterone levels and help in improving most of the adverse effects of hypogonadism. When clinical features suggest possible testosterone deficiency, the laboratory evaluation is initiated by measuring total testosterone, preferably in the morning [278,279]. The International Society for Sexual Medicine (ISSM) also recommends TRT for men if the total testosterone level is <8 nmol/L, or if the total testosterone level is <12 nmol/L and the patient is symptomatic, as long as maintenance of fertility is not desired [280]. According to the guidelines of the International Consultation for Sexual Medicine (ICSM), TRT is advisable when the level of total testosterone is <12 nmol/L or higher, based on clinical judgment [281] and/or in the presence of LOH symptoms, with these criteria being consistent across various countries. Testosterone is administered in the body through oral, buccal, intramuscular, subcutaneous, and transdermal routes [282,283]. Testosterone treatment, along with a lifelong physical exercise regimen, produces beneficial cardiovascular effects [284] and may help to maintain the normal level of testosterone in older men [285]. Although oral testosterone is available in India, Canada, Europe, and some other countries, it is not available in the USA [286]. Drugs containing PDE5 inhibitors, such as tadalafil, sildenafil and vardenafil, have been found to be effective in improving sexual function in men with ED [287,288,289] and work well in men older than 50 years as in younger men [145]. Other treatment strategies include X-ray visualization and penile implant surgery apart from androgen therapy [290,291].

Alternative treatment procedures, such as herbal medications, are also useful in managing ED [292]. Indian traditional system of medicine, Ayurveda, also offers various management options for hypogonadism or “AklalaJara” [293,294]. Fenugreek (*Trigonella foenum-graecum*) seeds have been considered to work well in cases of ED, male libido, sexual function, androgenic hormone level and can significantly decrease the total score for aging male syndrome (AMS). Roots of the age-old Ayurvedic herb “Ashwagandha” (*Withania somnifera*) is used to treat patients with low levels of testosterone and LH, semen volume, sperm count, sperm motility [295,296]. Other Indian traditional herbs used in Ayurveda and Unani systems of medicine, including *Asparagus racemosus*, *Mucuna pruriens*, *Tinospora cardifolia*, *Orchis latifolia*, may also help in combating aging, male reproductive tract disorder and helps in increasing quantity of semen as well as work as aphrodisiacs [297]. Herbs, such as “Tongkat Ali” (*Eurycoma longifolia*), are used for managing hypogonadism in Southeast Asian countries [295]. Studies on Japanese “Kampo” medicine, Korean herbal formulation “Ojayeonjonghwan” or KH-204, and Chinese herbal medicine saikokaryukotsuboreito (SKRBT) have reported their potential efficacy in managing hypogonadism [298,299,300]. Herbs, such as *Ginkgo biloba*, *Curcuma longa*, and *Camellia sinensis*, may also be useful in managing hypogonadism and ED [301,302]. Other herbal products from *Panax ginseng* and *Pausinystalia yohimbe* have also been used against ED [303]. Considering the above-mentioned environmental risk factors in the regular workup algorithm of clinicians may prove helpful in determining the root cause of hypogonadism and ED in such patients, who may be in need, by presenting a wider range of curative measures [80].

## 7. Conclusions

Proper functioning of the HPG axis is crucial for reproductive wellbeing. It is regulated by a complex interplay of neural, hormonal and metabolic signals, which may be disrupted by age-related hormone deficiency and environmental toxicants, including pesticides, radiations, air pollutants and plastic-originated agents and other endocrine-disrupting chemicals induced oxidative stress [44]. These disturbances may lead to impaired sexual potency, eventually causing disruption of psychological health in affected males. These environmental issues that may remain cryptic at times may become major mediators in the etiological context of LOH [80]. In conclusion, the present review attempts to identify the important environmental issues that may have a direct or indirect association with clinical hypogonadism and ED in men. The review also aims to incorporate the important environmental factors, such as pesticides, radiations, air pollution, plastic-originated agents, and other endocrine-disrupting chemicals, into the routine workup algorithm of clinicians to manage such patients, who may benefit.

## Figures and Tables

**Table 1:** Effects of pesticides, radiation, air pollution, agents originated in plastics and other endocrine-disrupting chemicals on hypogonadism and ED.

**Environmental Factor**	**Experimental Model**	**Experimental Type**	**Exposure Parameters**	**Findings**	**Comments**	**References**
Pesticides	Human	In vivo	Exposure to various pesticides for 2–11 years	Decreased serum testosterone level and LH	Decrease in sperm count and viability	[89]
	Rat	In vivo	Chlorpyrifos at 17.5 mg/kg bodyweight for 30 days	Decrease in serum testosterone level, sperm count and motility, but increase in cholesterol level	High cholesterol level in the testes decrease the androgen level and hampers spermatogenesis	[31]
	Rat	In vivo	Diazinon at 30 mg/kg body weight at 5 consecutive days for 30 days	Decrease in serum testosterone level	Reduction in sperm count, diameter of seminiferous tubules	[23]
	Rat	In vivo	12.5 mg/kg cypermethrin for 12 weeks	Decrease in serum testosterone level	Reduction in testicular weight, sperm count, viability, motility	[110]
Radiation	Human	In vivo	Ionizing radiation from external beam radiation therapy (EBRT) for 3 months, at the median 68Gy, as a part of prostate cancer treatment	Decrease in serum testosterone level	Suppression of spermatogenesis	[167]
	Rat	In vivo	Exposure to 900 MHz nonionizing radiation of cell phones	Increase in SOD activity	Production of ROS, lipid peroxidation, damaged spermatozoa	[24]
	Rat	In vivo	Exposure to 10 GHz non-ionizing radiation of XeThru X4 radar for 90 days	Decrease in serum testosterone and sex-hormone-binding protein	Effect on the male reproductive system	[150]
	Rat	In vivo	Exposure to 2.45 GHz of non-ionizing radiation	Decrease in serum testosterone level and increase in ROS, NO and MDA levels, expression of p53, Bax and active caspase in testes upregulated, while the expression of Bcl-xL, Bcl-2, procaspase and PARP-1 were downregulated	Decrease in seminiferous tubule diameter, sperm count, sperm motility and viability	[151]
	Rat	In vivo	Exposure to 7.5 Gy ionizing radiation for 5 days	Decrease in intracavernosal pressure	Reduced potential of attaining and maintaining prolonged penile erection	[170]
	Rat	In vivo	Exposure to 20 Gy ionizing radiation for 9 weeks	Decrease in intracavernosal pressure, increase in DNA oxidative stress in corpora cavernosa and prostate and increase in lipid peroxidation in corpora cavernosa	Erectile dysfunction may occur	[171]
	Rat	In vivo	Single dose of 4Gy ionizing radiation from X-rays	Decrease in serum testosterone level	Decrease in sperm count and motility, weight of testes, distortion in the architecture of seminiferous tubules	[172]
	Rat	In vivo	γ ionizing radiation	Decrease in serum testosterone level, SOD activity and a sharp rise in testicular MDA levels	Induction of oxidative stress	[173]
	Mice	In vivo	0.25 Gy ionizing radiation from X-ray twice a day for 4 days	Decrease in testosterone level, glutathione concentration and increase in ROS level, lipid peroxidation, serum LDH activity, antioxidant enzyme activities	Decrease in sperm count and motility. Testicular damage	[174]
Air Pollution	Rats	In vivo	6.5 mg/kg CdCl2 intraperitoneal injection for 5 days	Decrease in Testosterone level, antioxidant enzyme activities, PCNA antigen and increase in Cd concentration, lipid peroxidation, NO and MDA levels. Upregulation of BAX, TNFα factor, downregulation of BCL 2 gene	Decrease in weight of testes, depletion of DNA contents due to oxidative stress	[192]
	Rat	In vivo	4.28 mg/kg CdCl2 for 7 days	Decrease in serum testosterone concentration, SOD activity	Damage in the epithelium of seminiferous tubules	[193]
	Rat	In vivo	Single oral supplementation of 10 mg/kg/bodyweight of CdCl2	Decrease in serum testosterone level, increase in MDA level	Testicular damage due to Cd-induced oxidative stress	[194]
	Rat	In vivo	2.5 mg/kg/bodyweight oral supplementation of CdCl2	Decrease in serum testosterone level, FSH, LH level	Decrease in semen quality parameters and gonadosomatic index	[195]
	Rat	In vivo	Oral supplementation of 20 mg/kg PbAc for 10 days	Decrease in the levels of serum testosterone, FSH, LH levels, catalase activity and total antioxidant capacity. Increase in lipid peroxidation and levels of three lysosomal enzymes, including ACP, ß-NAG, and β-GAL in testes	Oxidative stress due to increase of ROS in testes. Accumulation of Pb in the testis tissues	[200]
	Rat	In vivo	0.1% PbAc in drinking water for 70 days	Decrease in serum testosterone level, SOD and glutathione peroxidase level	Reduction in weight of testes, the diameter of seminiferous tubules, epididymal sperm count	[201]
	Rat	In vivo	Oral supplementation of 50 mg/kg/bodyweight of Pb for 4 weeks	Decreased testosterone and GnRH level, glutathione, SOD, catalase activity	Imbalance in testosterone, GnRH levels and antioxidant enzymes can lead to male infertility	[202]
	Rat Leydig cell line R2C	In vitro	Cell lines incubated for 24 h in different concentration of Pb (50, 100, 200, 400 μM)	Decreased production of progesterone (precursor of testosterone), protein expression level of StAR, CYP11A1, 3β-HSD	Pb-induced oxidative stress can change the expression of antioxidant enzymes	[203]
	Rat	In vivo	Exposure to different concentration of PM2.5 once each week for 6 weeks	Ratio of intracavernosal pressure to mean atrial pressure decreased, ratio of smooth muscles to collagen decreased and ROS production increased	Testicular necrosis, hemorrhage, reduction in testicular size, degeneration of seminiferous tubules	[25]
	Mice	In vivo	Exposure to concentrated ambient PM2.5 (CAP)	Decrease in sperm count, circulating FSH and testosterone level, hypothalamic GnRH level	Adverse effects on testicular spermatogenesis resulting in sperm alterations	[217]
Other endocrine-disrupting chemicals	Human	In vivo	Exposure to BPA from working in factories	Decrease in testosterone level	Reduced sexual function, coitus frequency, inability to achieve an erection, ED	[235]
	Human	In vivo	Workers exposed to BPA in BPA and resin manufacturing companies	Lower sexual functions	Orgasmic function lowered leading to ED	[235]
	Human	In vivo	Exposure to BPA and BADGE)	Changes in endogenous sex hormone levels elevated urinary BPA concentration	Altered level of estrogen, androgen, gonadotropin, SHBG	[237]
	Human	In vivo	Exposure to DBP and DEHP in polyvinyl chloride flooring producing factory	High levels of MBP and MEHP in body and decrease in testosterone, FSH, LH level.	Reduction in steroidogenic activity and spontaneous erection that might lead to ED.	[232]
	Human	In vivo	Exposure to PFAS	Negative association between PFOS with testosterone	Decrease in the testosterone level with no effect on semen quality	[254]
	Rat	In vivo	Administration of DEHP for 30 days	Affects testicular physiology and testosterone production	Deformation of seminiferous tubules along with an increase in Leydig cell number	[242]
	Rat	In vivo	Exposure of Leydig cells to different concentrations of PCB	Inhibition of basal and LH-stimulated testosterone production	Testosterone level decreases with decreased the activity of steroidogenic enzymes, enzymatic and nonenzymatic antioxidants	[262]
	Mice Leydig cell line TM3	In vitro	DEHP treatment to Leydig cell TM3 for 24 h	Disturbance in the HPG axis	Reduction in LH and FSH level as well as testosterone level	[242]

FSH: follicle-stimulating hormone, LH: luteinizing hormone, EBRT: external beam radiation therapy, SOD: superoxide dismutase, ROS: reactive oxygen species, NO: nitric oxide, MDA: malondialdehyde, Bcl-xL: B-cell lymphoma-extra-large, Bcl-2: B-cell lymphoma 2, PARP-1: poly-[ADP-ribose] polymerase 1, LDH: lactate dehydrogenase, PCNA: proliferating cell nuclear antigen, BAX: Bcl-2 associated X protein, TNF-α: tumor necrosis factor-α, ACP: acyl carrier protein, β-NAG: β N-acetyl glucosamine, β-GAL: β galactosidase, GnRH: gonadotropin-releasing hormone, PM: particulate matter, BPA: bisphenol A, BADGE: BPA diglycidyl ether, DBP: dibutyl phthalate, DEHP: diethyl hexyl phthalate, MBP: mono butyl phthalate, MEHP: mono ethyl hexyl phthalate, SHBG: sex-hormone-binding protein, ED: erectile dysfunction, PFAS: perfluoroalkyl substances, PFOS: perfluorooctanesulfonic acid, PCB: polychlorinated biphenyl, HPG: hypothalamic–pituitary–gonadal.
